# Are CONSORT checklists submitted by authors adequately reflecting what information is actually reported in published papers?

**DOI:** 10.1186/s13063-018-2475-0

**Published:** 2018-01-29

**Authors:** David Blanco, Alice M. Biggane, Erik Cobo, MiRoR network, Doug Altman, Lorenzo Bertizzolo, Isabelle Boutron, Efstathia Gkioni, Ketevan Glonti, Jamie Kirkham, Camila Olarte, Maria Olsen, Cecilia Superchi

**Affiliations:** 1 Statistics and Operations Research Department, Barcelona Tech, Barcelona, Spain; 2 Department of Biostatistics, University of Liverpool, Liverpool, UK; 3 INSERM, U1153 Epidemiology and Biostatistics Sorbonne Paris Cité Research Center (CRESS), Methods of Therapeutic Evaluation of Chronic Diseases (METHODS) Team, Paris Descartes University, Sorbonne Paris Cité, Paris, F-75014 France

**Keywords:** Reporting guidelines, Completeness of reporting, Reporting inconsistencies, CONSORT, Endorsement

## Abstract

**Background:**

Compulsory submission of a checklist from the relevant reporting guideline is one of the most widespread journal requirements aiming to improve completeness of reporting. However, the current suboptimal levels of adherence to reporting guidelines observed in the literature may indicate that this journal policy is not having a significant effect.

**Findings:**

We explored whether authors provided the appropriate CONSORT checklist extension for their study and whether there were inconsistencies between what authors claimed on the submitted checklist and what was actually reported in the published paper. We randomly selected 12 randomized trials from three journals that provide the originally submitted checklist and analyzed six core CONSORT items. Only one paper used the appropriate checklist extension and had no inconsistencies between what was claimed in the submitted checklist and what was reported in the published paper.

**Conclusion:**

Journals should take further actions to take full advantage of the requirement for the submission of fulfilled CONSORT checklists, thus ensuring that these checklists reflect what is reported in the manuscript.

**Electronic supplementary material:**

The online version of this article (10.1186/s13063-018-2475-0) contains supplementary material, which is available to authorized users.

## Background

Completeness of reporting is a critical issue in health research. It enhances the transparency of research methods and findings, thus promoting their credibility and reproducibility. In an attempt to improve completeness of reporting, several reporting guidelines for different study designs and clinical areas were developed in the last two decades [1]. The Consolidated Standards of Reporting Trials (CONSORT), was created in 1996 to help authors report the methods and findings of randomized trials [2]. It has been revised and updated twice [3, 4]. ‘Endorsement’ of CONSORT by medical journals has been one of the most widespread actions implemented to improve the levels of completeness of reporting of randomized trials. It is defined as any of the following situations: (a) journal editorial statement endorsing CONSORT; (b) requirement or recommendation in journal’s instructions to authors to follow CONSORT when preparing their manuscript; or (c) requirement for authors to submit a CONSORT checklist with their manuscript [5]. Existing evidence shows that, despite modest improvements when CONSORT is endorsed by journals, the completeness of reporting of trials remains suboptimal [5].

In recent years, dozens of medical journals have opted for policy (c) in the previous paragraph, as it has the most potential to improve completeness of reporting. In addition, in an effort to make the editorial process more transparent and credible, some journals following this policy, such as *PLoS One*, *BMJ Open* and *Trials*, also make the original CONSORT checklist submitted by the authors accessible for their readers as supplementary material. However, the current suboptimal levels of adherence to reporting guidelines observed in the literature across different research areas and study designs [6] may indicate that this journal policy is not having the desired effect. To date, there has been no investigation into whether or not there are inconsistencies between what authors claim to have reported in the submitted checklist and what is actually reported in the published paper.

A number of specific study designs (such as cluster designs) or interventions (such as non-pharmacological interventions) have additional specific extensions [7, 8]. In the journals mentioned, authors are required to submit the CONSORT extension that applies to their study. Thus, it is essential to assess whether authors actually provide the appropriate extensions for their studies.

In this commentary, we explore (1) whether authors provide the appropriate CONSORT checklist and (2) whether there are inconsistencies between what authors claim to have reported in the submitted checklist and what they have actually reported in the published paper, for those papers submitted with the appropriate checklist. Our intention is to illustrate whether CONSORT checklist fulfilment by authors should be considered a guarantee that CONSORT items are effectively satisfied.

## Our findings

We looked at 12 randomly selected randomized trials published in either *Trials*, *BMJ Open* or *PLoS One* between 1 January 2016 and 30 June 2017 (see Additional file 1: Point 1, search strategy and study selection). We chose those journals because they require authors to submit a CONSORT checklist with their manuscript and make this checklist accessible for their readers as supplementary material. For each paper selected, we retrieved the initial CONSORT checklist and manuscript submitted by the authors. First, we independently determined whether the CONSORT checklist originally submitted by the authors was the appropriate extension for the study design. Then, for papers using the appropriate checklist, we compared it with what was actually reported in the published paper to identify any inconsistencies (see Additional file 1: Point 2, analysis of inconsistencies). We focused on six core CONSORT items of the ‘Methods’ and ‘Results’ sections: (6a) outcomes; (8a) randomization or sequence generation; (9) allocation concealment mechanism; (11a) blinding; (13a) flow of participants and (13b) losses and exclusions. We selected those items because they are essential for systematic reviewers to evaluate the risk of bias. For each item, the CONSORT explanation and elaboration document [9] was used to determine what information should be reported.

We graded the consistency between what authors said and what they reported for each item as follows:

- *Completely consistent*: There was no divergence between what authors claimed to have reported through the originally submitted CONSORT checklist and what they reported in the published paper.
- *Partially consistent*: This may include either (a) partial absence of relevant information that was expected to be reported or (b) information corresponding to that item was reported in a different part of the paper from that specified in the checklist.
- *Not consistent*: Authors claimed to have reported that item through the CONSORT checklist but did not adequately report the information in the published paper.

From the 12 randomly selected randomized trials, the standard CONSORT checklist was appropriate for six papers (four of which were standard parallel trials covered by CONSORT and two of which were crossover trials, for which there is not an extension yet). The other six required CONSORT extensions (for cluster trials, three; for pragmatic trials, two; and for non-pharmacological interventions, one) but authors did not use them in any case, despite the extensions being available at the time of submission. The aforementioned extensions were published between 2008 and 2012 [7, 8, 10], yet the six papers requiring their uptake were all submitted later than May 2015.

**Fig. 1:**
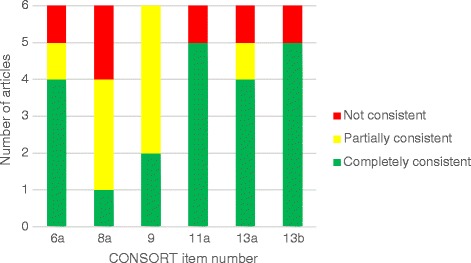
Reporting inconsistencies found for the six papers that used the appropriate CONSORT checklist

For the six papers for which an appropriate CONSORT checklist was submitted, only one paper had complete consistency between the checklist and the published paper. The most concerning problems centred on items 8 and 9 (see Fig. 1). For example, a possible inconsistency identified regarding CONSORT item 9 (Allocation concealment mechanism – mechanism used to implement the random allocation sequence, describing any steps taken to conceal the sequence until interventions were assigned) is the following: authors claim through the checklist that they have reported the item; however, we found that the paper stated “The sequence of the test conditions was randomized for each participant by LB and KDK. A card was made for each possible sequence and a card was picked blindly for each participant.” This statement does not make it clear how the authors implemented the random allocation sequence nor how they kept the assignment concealed. Picking a card does not guarantee that allocation used in the analysis has preceded treatment, nor does it allow readers to reproduce the mechanism used to implement the random allocation sequence.

A full summary of the evaluations for all six items across all six papers is shown in Additional file 2. The level of reporting inconsistencies found for every item among the six papers considered in the analysis is provided in Fig. 1. Illustrative examples of inconsistencies found for each item are shown in Additional file 3.

## Why could reporting inconsistencies occur?

Among the numerous potential reasons for the presence of reporting inconsistencies, we underline two explanations. Firstly, it is possible that authors are not attentive to the requirements of CONSORT or, despite their efforts to be compliant with the requirements, they are struggling to interpret certain items or the level of detail that is required. Examples include:

- *Item 8a* ‘Method used to generate the random allocation sequence’. This item was adequately reported in just one of the papers screened. Within this item, there were various reasons for the inconsistencies, including: lack of thorough and complete reporting from the authors (see example for this item in Additional file 3) and the nontechnical use of the term ‘random’ (‘The study nurse randomly opened a preformed envelope containing the allocated treatment regimen’).
- *Item 11a* ‘If done, who was blinded after assignment to interventions (for example, participants, care providers, those assessing outcomes) and how’. The initial phrase ‘if done’ may have caused confusion about whether or not the authors have to report what groups of individuals involved in the trial were unblinded. To avoid authors not disclosing the lack of blinding if the trial was unmasked, we suggest that future versions of the CONSORT checklist delete the phrase ‘if done’.

The misinterpretation of CONSORT is a major concern, as it means that essential information regarding study conduct is miscommunicated. This is particularly relevant for item 11a, as according to PRISMA item 19 [11], when assessing the risk of bias of a study it is necessary to know whether or not patients, health care providers, data collectors and outcome assessors are blinded.

Secondly, the issues described in this study might also lie with the reviewers and editors. It is possible that they are falsely reassured with regard to the reporting quality of the manuscripts, merely by the presence of a completed checklist. Moreover, the fact that the reporting inconsistencies persist throughout the editorial process might mean that editors and reviewers are not using reporting guidelines as a method to review manuscripts [12] although the CONSORT explanation and elaboration document suggests that: ‘Readers, peer reviewers and editors can also use CONSORT to help them critically appraise and interpret reports of randomized controlled trials.’

## Possible solutions

In an effort to take full advantage of requiring the submission of checklists, journals should consider clarifying their stance on whether the full checklists, or at least the checklists’ core items should be examined by editors or reviewers, or even by trained editorial assistants [13].

As the page numbers reported by authors in the checklist are not updated after the peer review process and the typesetting process, they do not correspond to the page where the information is placed in the published paper. Having to update the page numbers in the checklist from original submission to published paper could act as a checkpoint for editors or reviewers to remind them to verify whether authors are appropriately reporting the key information in the latest version of the manuscript. An alternative solution could be to ask authors to address the section and the paragraph where the information corresponding to each item is reported. This would reduce the risk of overburdening the authors and could potentially help deter the misconception that these checklists are merely bureaucratic. Furthermore, making available the updated checklist could help systematic reviewers easily and quickly find the relevant information to assess the risk of bias of the studies included in the reviews [11].

## Conclusions

Poor-quality reporting in health research critically affects the credibility, reproducibility and generalizability of the methods and findings of randomized trials. For these reasons, further exploration of methods that will obligate authors to consistently and accurately fulfil and submit CONSORT checklists is required. Moreover, journals should consider making clear whether the checklists should be examined by editors, peer reviewers or a trained editorial assistant.

## Additional files

Additional file 1: Search strategy, study selection and analysis of inconsistencies. (DOCX 15 kb)

Additional file 2: Summary of evaluations. (XLSX 77 kb)

Additional file 3: Examples of inconsistencies. (DOCX 15 kb)

## Abbreviations

CONSORT: Consolidated Standards of Reporting Trials
MiRoR: Methods in Research on Research
PRISMA: Preferred Reporting Items for Systematic Reviews and Meta-Analyses
