# Supplementary material for: Are CONSORT checklists submitted by authors adequately reflecting what information is actually reported in published papers?
Source: Trials. 2018 Jan 29;19:80. doi: 10.1186/s13063-018-2475-0 (PMC5789595; doi:10.1186/s13063-018-2475-0)
Supplement: Supplementary file 1 — Search strategy, study selection and analysis of inconsistencies. (DOCX 15 kb) [file 13063_2018_2475_MOESM1_ESM.docx]

1. **Search strategy and study selection**

PubMed was searched on 12/06/2017 for randomised trials published in PLOS One, BMJ Open, and Trials between 01/01/2016 and 01/06/2017 (see Box 1). After filtering the results by “Randomised Controlled Trial”, the search returned 232 hits (176 from Plos ONE, 36 from BMJ Open, and 20 from Trials). R was used to generate three sequences of random numbers between 0 and 1 of size 176, 36, and 20 (see Box 2). From each of the three journals five papers were selected by matching the positions of the five highest numbers in each of the sequences with the positions of the PubMED search results ordered by “Journal”. Some of the papers initially selected were not suitable for further analysis because either they were not RCTs or the CONSORT checklist was not available. Therefore, those papers were excluded and new ones were randomly selected in Plos ONE and BMJ Open papers until five papers fulfilling the inclusion criteria were found for each journal. For Trials, only two papers meeting the inclusion criteria were found among the twenty papers available, since the others were either not RCTs or the checklist was not available. Therefore, the final analysis included twelve papers.

1. **Analysis of inconsistencies**

The twelve selected papers were randomly split into three groups of four papers. Each of the groups was reviewed by one of the three main authors (AB, DB, and EC) and two of six selected researchers (LB, EG, KG, CO, MO, and CS) from the Methods in Research on Research (MiRoR) Network (<http://miror-ejd.eu/>). Disagreements were resolved through discussion.

**Box 1: Search terms for MEDLINE (from January 1, 2017, to June 6, 2017) via PubMed.**

| **Steps** | **Search terms** |
| --- | --- |
| S1 | Trials [Journal] |
| S2 | BMJ Open [Journal] |
| S3 | Plos ONE [Journal] |
| S4 | S1 **OR** S2 **OR** S3 **NOT** Protocol |
| S5 | S4 **AND** Randomized Controlled Trial[ptyp] **AND** ("2017/01/01"[PDAT] : "2017/06/06"[PDAT]) |

**Box 2: R commands for randomly selecting papers from PLOS One, BMJ Open, and Trials**

randBMJOpen=runif(36)

positionsBMJOpen=order(randBMJOpen)

randPlosONE=runif(176)

positionsPlosONE=order(randPlosONE)

randTrials=runif(20)

positionsTrials=order(randTrials)
