# Supplementary material for: Are CONSORT checklists submitted by authors adequately reflecting what information is actually reported in published papers?
Source: Trials. 2018 Jan 29;19:80. doi: 10.1186/s13063-018-2475-0 (PMC5789595; doi:10.1186/s13063-018-2475-0)
Supplement: Supplementary file 3 — Examples of inconsistencies. (DOCX 15 kb) [file 13063_2018_2475_MOESM3_ESM.docx]

**Illustrative examples of inconsistencies found for each item**

| **CONSORT item claimed to be reported** | **Article**  **number** | **Information reported in the final article** | **Evaluation of the item** | **Evaluation rationale** |
| --- | --- | --- | --- | --- |
| **6a:** Outcomes- Completely defined pre-specified primary and secondary outcome measures, including how and when they were assessed. | 10 | *No information related to this item is visibly reported where referenced.* | Not consistent | Firstly, the outcomes are not clearly specified. Secondly, while the authors say in the abstract “Denture biofilm coverage was scored”, this outcome is not mentioned in the location referenced in the checklist. |
| **8a:** Sequence generation - Method used to generate the random allocation sequence. | 9 | “Participants were randomly assigned to one of two parallel groups, in a 1:1 ratio”. | Not consistent | The method used to generate the random allocation sequence is not explicitly mentioned. |
| **9:** Allocation concealment mechanism - Mechanism used to implement the random allocation sequence (such as sequentially numbered containers), describing any steps taken to conceal the sequence until interventions were assigned. | 10 | “The sequence of the test conditions was randomized for each participant by LB and KDK. A card was made for each possible sequence and a card was picked blindly for each participant.” | Partially consistent | It is not clear how the authors implemented the random allocation sequence nor how they kept the assignment concealed. Picking a card does not guarantee that allocation used in the analysis has preceded treatment, neither allows readers to reproduce the mechanism used to implement the random allocation sequence. |
| **11a:** Blinding - If done, who was blinded after assignment to interventions (for example, participants, care providers, those assessing outcomes) and how. | 8 | “Patients were randomized by the study nurse, blinded from both the investigator and study participant”. | Not consistent | No information is provided about the degree of blinding of care providers and those assessing the outcomes. |
| **13a:** Participant flow- For each group, the numbers of participants who were randomly assigned, received intended treatment, and were analysed for the primary outcome. | 11 | In the flow diagram: “Allocated to intervention (n=515) → Follow-up observations (n=925) - Study termination/Lost in follow up (n=364)”. | Not consistent | N is used for two different units: observations and participants. Moreover, authors report two different reasons simultaneously: study termination and lost to follow up. |
| **13b:** Participant flow- For each group, losses and exclusions after randomisation, together with reasons. | 9 | In the text: “In the exercise condition, four participants dropped out during the intervention: reasons were injuries (n = 3) and migration (n = 1)”. In the flow diagram: “Allocated to exercise intervention (n=50) - Received allocated intervention (n=50) → Analysed: post-intervention (n=49) - follow-up (n=48). | Not consistent | The lost to follow up numbers reported are different in the text and the flow diagram. |
